# Supplementary material for: Projection neurons in Drosophila antennal lobes signal the acceleration of odor concentrations
Source: eLife. 2015 May 14;4:e06651. doi: 10.7554/eLife.06651 (PMC4466247; doi:10.7554/eLife.06651)
Supplement: Source code 1. — Custom software written in MATLAB for the analysis of OSN and PN data. DOI: http://dx.doi.org/10.7554/eLife.06651.010 [file elife06651s001.zip › 20101016FlySpikes_Tutorial.pdf]

# FlySpikes 1.0 - User Manual and Tutorials

Anmo Kim (ak2263@columbia.edu)

2006-2010

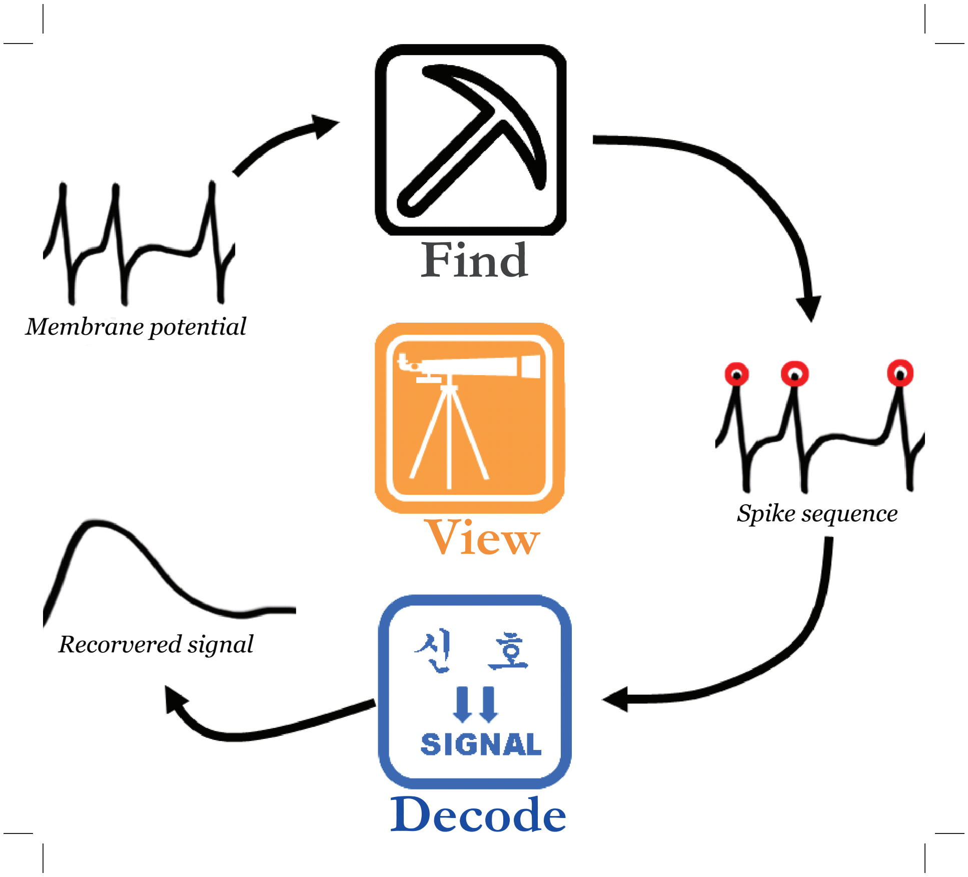

# Contents

**1 Introduction** **3**

**2 Installation of *FlySpikes 1.0* and Sample Data** **3**

**3 Tutorial - plotting triangle waveforms and responses** **4**

**4 Tutorial - plotting frozen white noise waveforms** **7**

**5 Tutorial - spike-triggered average (STA) from 150 white noise waveforms** **10**

# 1 Introduction

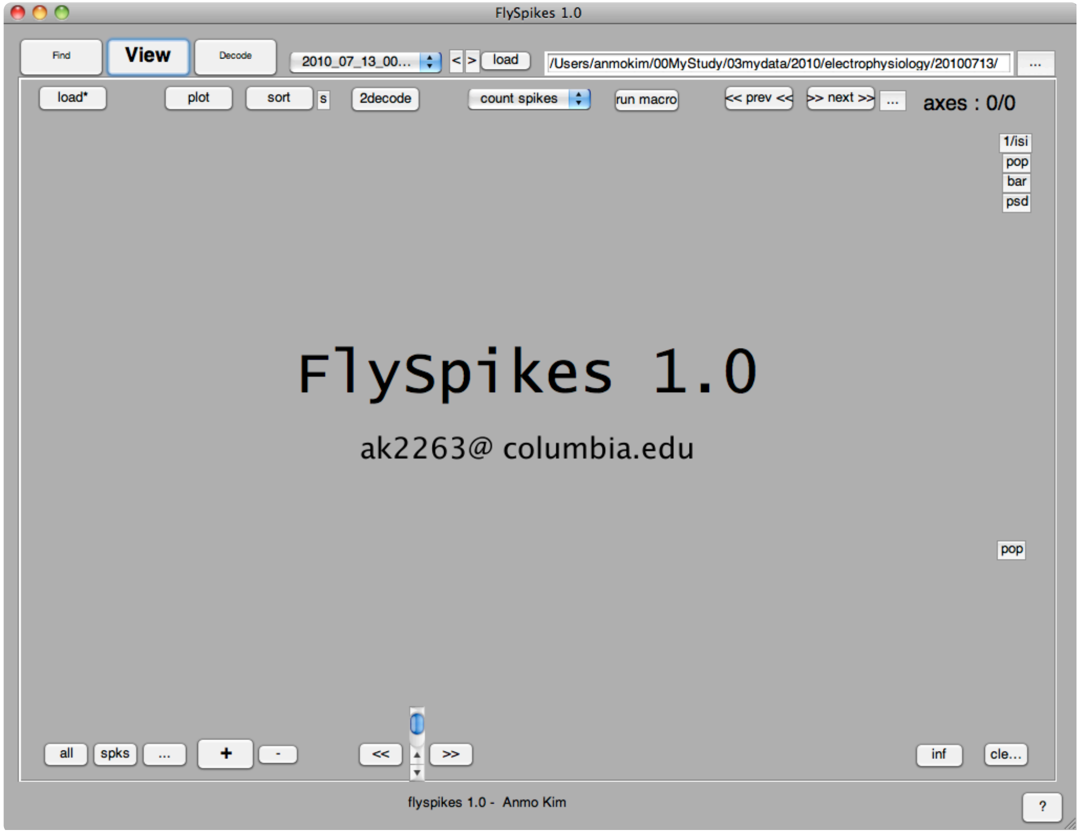


# 2 Installation of *FlySpikes 1.0* and Sample Data

First, include the main folder of the FlySpikes project to the MATLAB path, and run the FlySpikes by typing flyspikes at the command prompt.

# 3 Tutorial - plotting triangle waveforms and responses

In this section, we will provide a tutorial session in which a set of triangle odor concentration waveform and the corresponding PSTHs are plotted. The 18 sample data files are provided in the *Tutorial1* folder. Before starting the following tutorial instructions, make sure the current folder in ***FlySpikes*** is the *Tutorial1* folder. (If you cannot find the folder, please download *Tutorial1.zip* and extract it to your computer and move to the extracted *Tutorial1* folder using ... button next to the path information at the top right corner.

- Selecting data files to be loaded

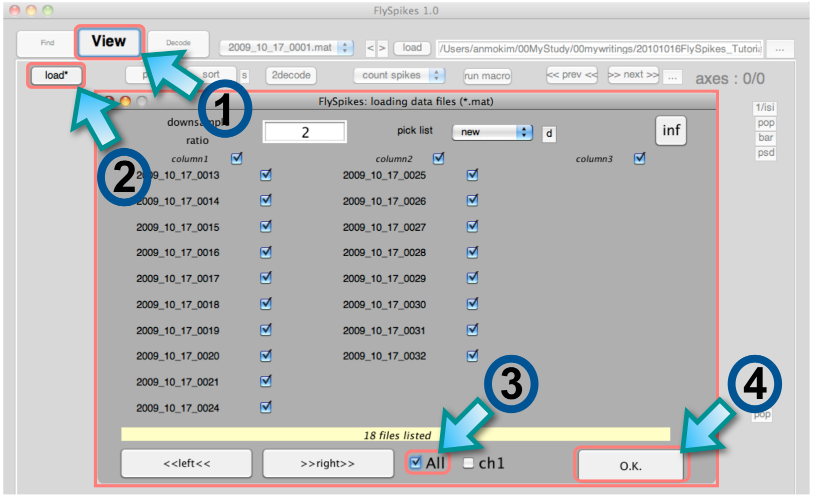


1. move to the *View* tab
2. click *load** button to open the loading modal window
3. check the *All* checkbox to select all the data files.
4. click *O.K.* button to complete the selection of the data files to be loaded

- Browsing among the loaded data files

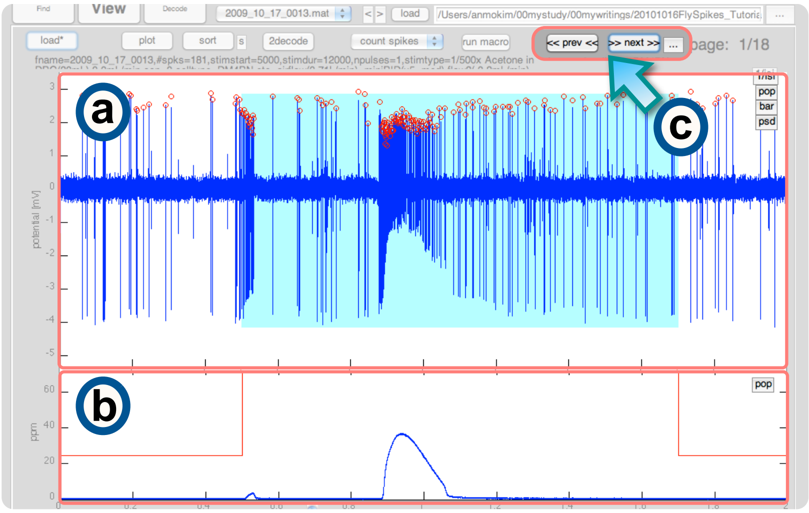


a. a neuronal output voltage (or current) signal is displayed on the top axes

b. if available, an input stimulus is displayed on the bottom axes

c. use *<<left<<* or *>>right>>* buttons to browsing among loaded pages. the ... button allows one to jump to a page by providing its index

- Plotting all individual odor waveforms

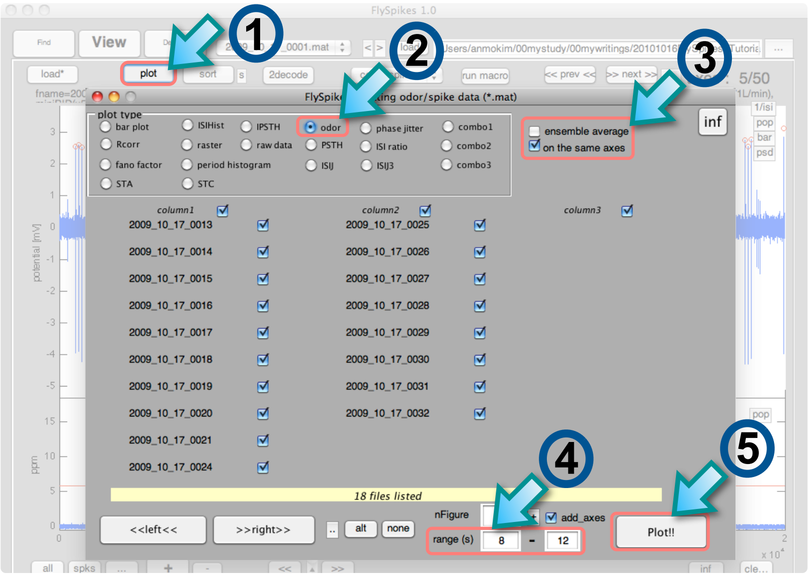


1. click *plot* to open the plotting modal window
2. choose *odor* to plot odor waveforms
3. uncheck *ensemble average* and check *on the same axes*
4. set the plotting range from 8 s to 12 s
5. click the *Plot!!* button

Result: odor waveforms 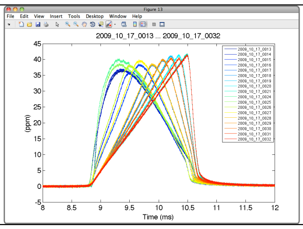 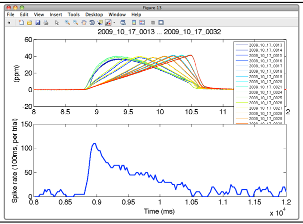 Result: a PSTH

- Plotting a PSTH for the left-most triangle odor waveforms

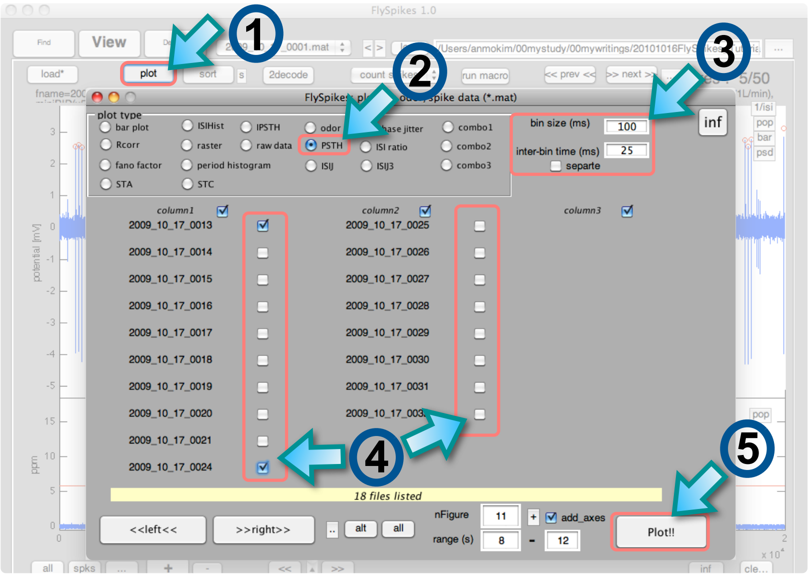


1. click *plot* to open the plotting modal window
2. choose *PSTH*
3. set the *bin size* to 100 ms and the *inter-bin time* to 25 ms. uncheck the *separate* checkbox
4. choose only xxx_0013 and xxx_0024
5. click the *Plot!!* button

- Plotting input/output pairs of the loaded data files

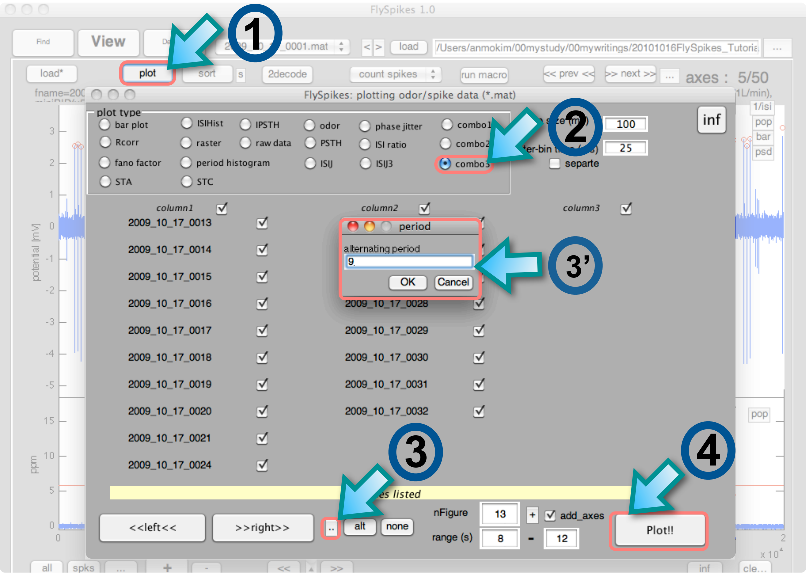


1. click *plot* to open the plotting modal window
2. choose *combo3* to plot both input and output
3. click ... button to set the period. (3') type *9* and click *OK*
4. click the *Plot!!* button

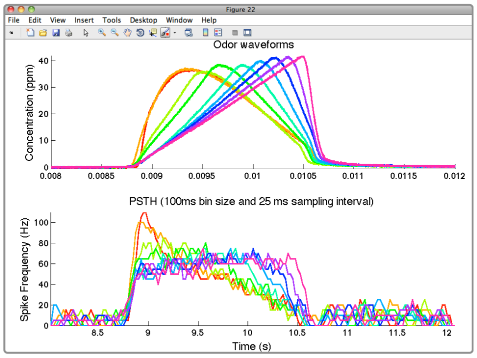


< Result: Nine input/output pairs >

- Some information about this tutorial
  - The data in this tutorial is a part of the data used in [1] to plot Figure 3. The experiment was carried out by Yevgeniy Slutskiy.
  - *combo3* plots average odor waveforms and the corresponding PSTHs of a set of data files
  - plotting sets for *combo3* are determined by the alternating period (... next to *alt*); i.e., when the alternating period is $N$, $k$th data file belongs to the ($k$ mod $N$)th set. Unchecked data files are counted when determining the set, but skipped for actual plotting.
  - *combo3* saves the resulting plots as matlab figures and the input/output pairs as a .mat file in *Tutorial1/doc* folder; for example, 2009_10_17_0001_0054_odor_psth_100_25.mat is created once this tutorial is executed.

# 4 Tutorial - plotting frozen white noise waveforms

In this section, we will provide a tutorial session that will generate a figure of frozen noise odor waveforms, the corresponding spike rasters, and the PSTH. 59 sample data files are provided in the *Tutorial2* folder. Before starting the tutorial instructions, make sure the current folder in ***FlySpikes*** is the *Tutorial2* folder. (If you cannot find the folder, please download *Tutorial2.zip* and extract it to your computer and move to the extracted *Tutorial2* folder using ... button next to the path information at the top right corner.

- Selecting data files to be loaded

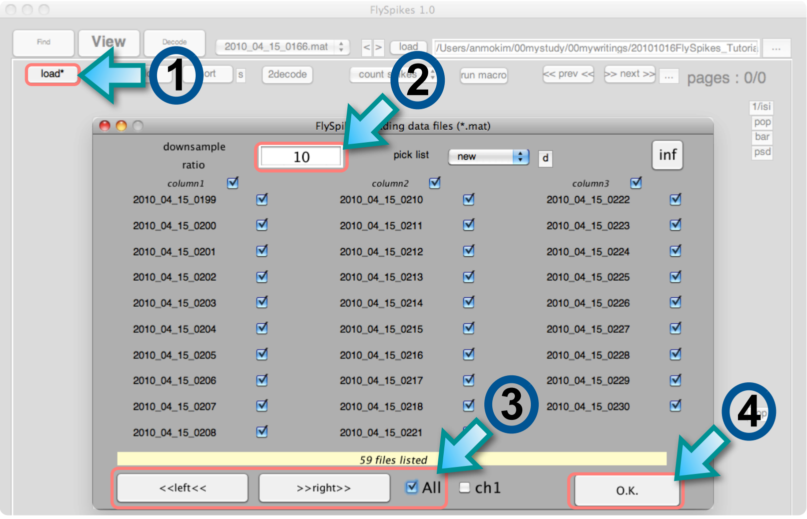


1. click *load**
2. set the *downsample ratio* as *10 to plot the input/output after downsampling (this reduces the memory usage)*
3. *using <<left<<, >>right>>, and All, select all 59 files*
4. *clikc O.K.*

- Plotting all individual frozen noise odor waveforms

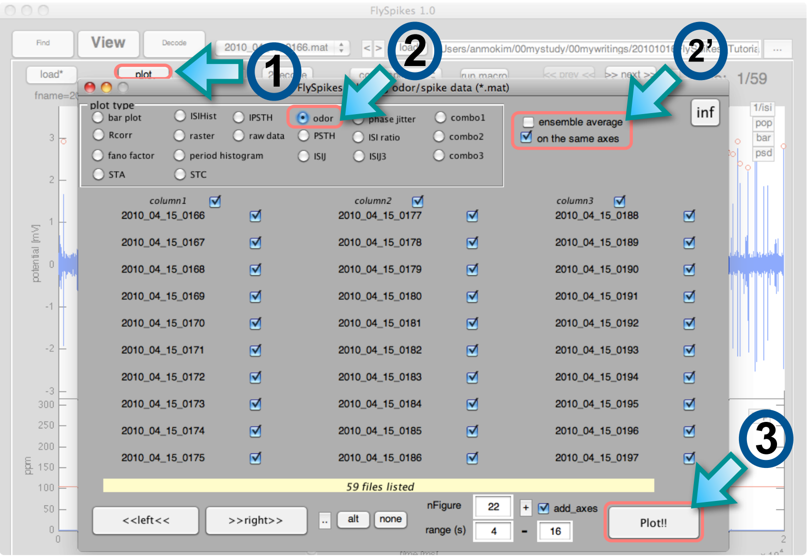


1. click *plot* in the *View* tab
2. select *odor*. (2') uncheck *ensemble average*, and check *on the same axes*
3. click *Plot!!*

- Plotting all individual rasters

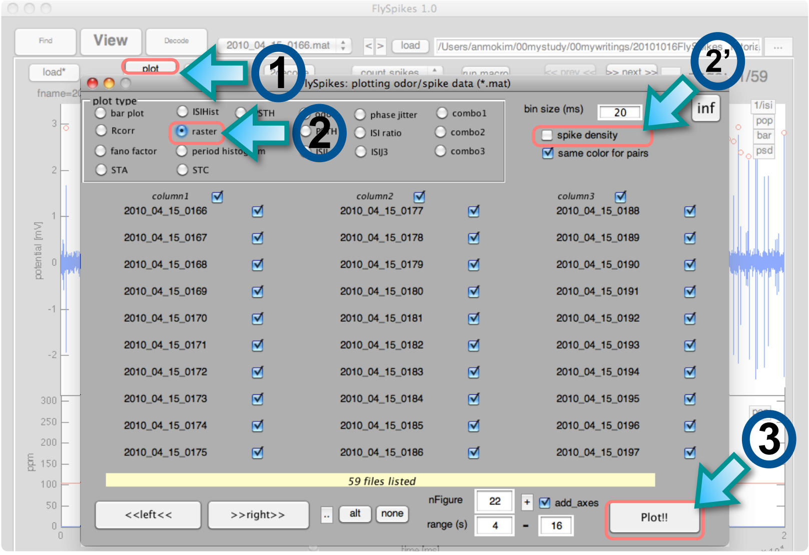


1. click *plot* in the *View* tab
2. select *raster*. (2') uncheck *spike intensity*
3. click *Plot!!*

- Plotting a PSTH

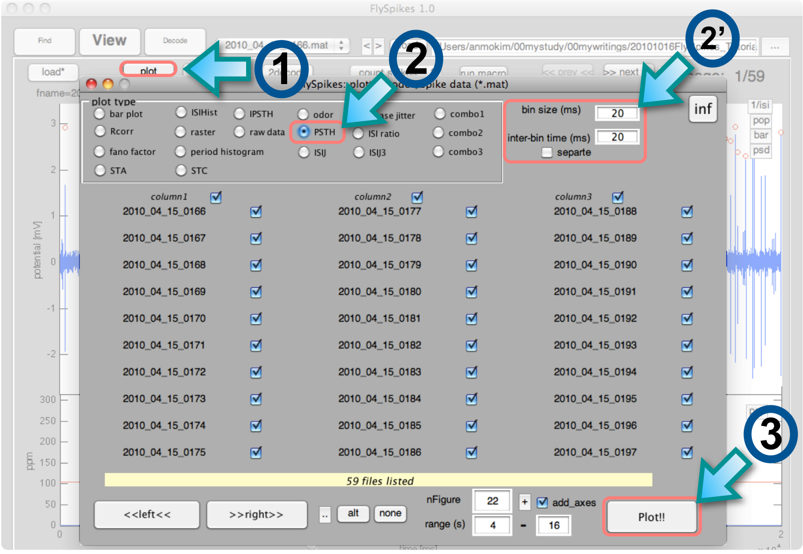


1. click *plot* in the *View* tab
2. select *PSTH*.

   (2') uncheck *separate*, set both *bin size* and *inter-bin size* as 20 ms
3. click *Plot!!*

- Result

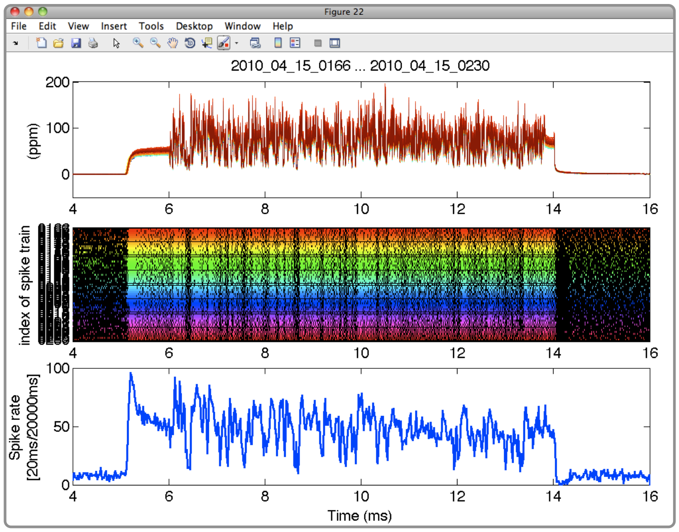


- Some information about this tutorial
  - The data used in this tutorial is a part of the data used in [1] to plot Figure 4. The experiment was carried out by Yevgeniy Slutskiy.
  - The *downsampling ratio* is applied only to the graphics (plots) and does not change the sampling interval of the internal data used by ***FlySpikes***

# 5 Tutorial - spike-triggered average (STA) from 150 white noise waveforms

In this section, we will provide a tutorial session that will compute a spike-triggered average from 150 white noise odor waveforms. The data files are provided in the *Tutorial3* folder. Before starting the tutorial instructions, make sure the current folder in ***FlySpikes*** is the *Tutorial3* folder. (If you cannot find the folder, please download *Tutorial3.zip* and extract it to your computer and move to the extracted *Tutorial3* folder using ... button next to the path information at the top right corner.

- Selecting data files to be loaded

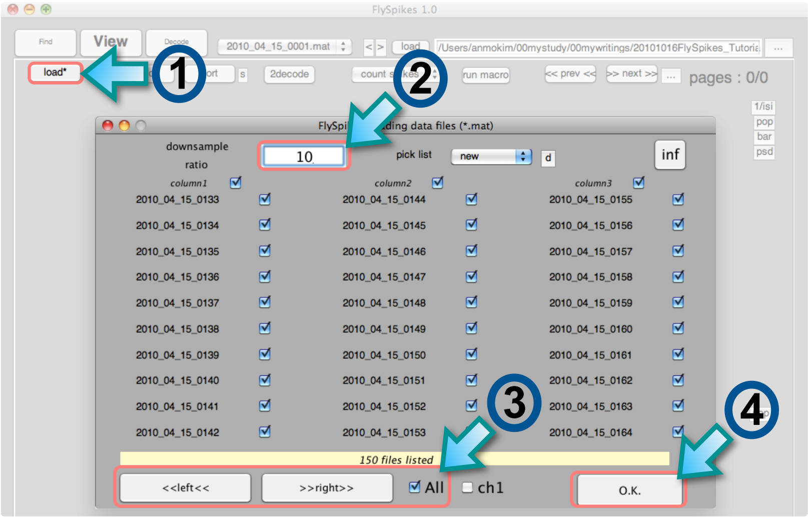


1. click *load**
2. set the *downsample ratio* as *10 to plot the input/output after downsampling (this reduces the memory usage)*
3. *using* *<<left<<*, *>>right>>*, *and* *All*, *select all 150 files*
4. *clikc* *O.K.*

- Computing and plotting the spike-triggered average

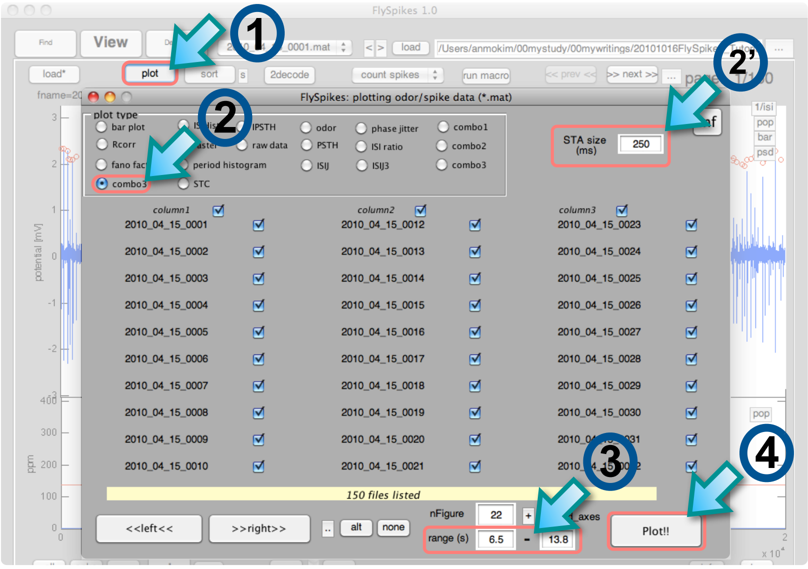


1. click *plot* in the *View* tab
2. select *STA*. (2') set *the STA size* as 250 ms
3. set the range from 6.5 s to 13.8 s
4. click *Plot!!*

- Result

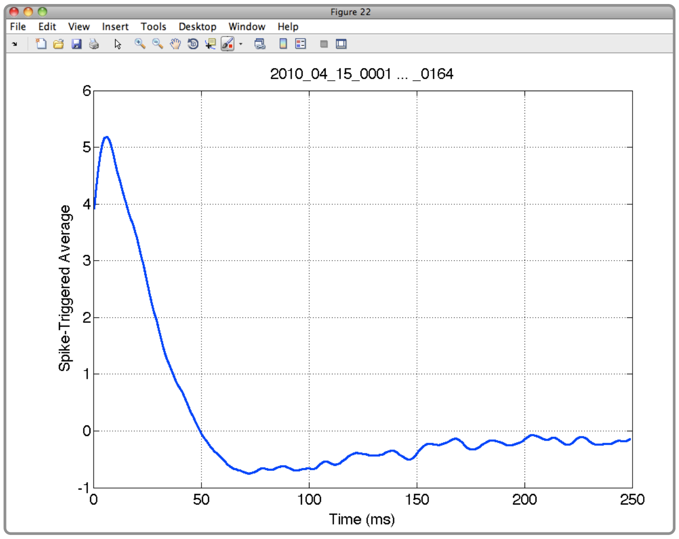


- Some information about this tutorial
  - The data used in this tutorial is a part of the data used in [1] to plot Figure 5. The experiment was carried out by Yevgeniy Slutskiy.
  - *STA* saves the spike-triggered average as well as the spike-triggered ensemble as a .mat file in *Tutorial1/doc* folder (this tutorial will create 2010_04_15_0001__0164_sta_250.mat).

# References

[1] Anmo J Kim, Aurel A Lazar, and Yevgeniy B Slutskiy. System identification of drosophila olfactory sensory neurons. *J Comput Neurosci*, Aug 2010.
